# The effect of *Alnus incana* (L.) Moench extracts in ameliorating iron overload-induced hepatotoxicity in male albino rats

**DOI:** 10.1038/s41598-023-34480-6

**Published:** 2023-05-11

**Authors:** Fatma Abo-Elghiet, Shaza A. Mohamed, Noha A. E. Yasin, Abeer Temraz, Walid Hamdy El-Tantawy, Samah Fathy Ahmed

**Affiliations:** 1 Pharmacognosy and Medicinal Plants Department, Faculty of Pharmacy for Girls, Al Azhar University, Cairo, Egypt; 2 Cytology and Histology Department, Faculty of Veterinary Medicine, Cairo University, Giza, Egypt; 3 National Organization for Drug Control and Research, Dokki, Cairo, Egypt

**Keywords:** Toxicology, Hepatotoxicity, Natural products

## Abstract

Iron overload causes multiorgan dysfunction and serious damage. *Alnus incana* from the family Betulaceae, widely distributed in North America, is used for treating diseases. In this study, we investigated the iron chelating, antioxidant, anti-inflammatory, and antiapoptotic activities of the total and butanol extract from *Alnus incana* in iron-overloaded rats and identified the bioactive components in both extracts using liquid chromatography-mass spectrometry. We induced iron overload in the rats via six intramuscular injections of 12.5 mg iron dextran/100 g body weight for 30 days. The rats were then administered 60 mg ferrous sulfate /kg body weight once daily using a gastric tube. The total and butanol extracts were given orally, and the reference drug (deferoxamine) was administered subcutaneously for another month. After two months, we evaluated the biochemical, histopathological, histochemical, and immunohistochemical parameters. Iron overload significantly increased the serum iron level, liver biomarker activities, hepatic iron content, malondialdehyde, tumor necrosis factor-alpha, and caspase-3 levels. It also substantially (*P* < 0.05) reduced serum albumin, total protein, and total bilirubin content, and hepatic reduced glutathione levels. It caused severe histopathological alterations compared to the control rats, which were markedly (*P* < 0.05) ameliorated after treatment. The total extract exhibited significantly higher anti-inflammatory and antiapoptotic activities but lower antioxidant and iron-chelating activities than the butanol extract. Several polyphenolic compounds, including flavonoids and phenolic acids, were detected by ultraperformance liquid chromatography-electrospray ionization-quadrupole time-of-flight mass spectrometry (UPLC-ESI-QTOF-MS) analysis. Our findings suggest that both extracts might alleviate iron overload-induced hepatoxicity and other pathological conditions characterized by hepatic iron overload, including thalassemia and sickle-cell anemia.

## Introduction

Iron overload toxicity has been linked to hereditary hemochromatosis, thalassemia, and liver diseases, including chronic and alcoholic hepatitis^1^. Most patients with homozygous β-thalassemia have severe progressive anemia requiring regular vital blood transfusions, although a few remain transfusion-independent^2^. Because of chronic transfusion and increased gastrointestinal absorption, iron accumulates in many organs and tissues, leading to progressive multiorgan dysfunction including the liver, heart, and endocrine glands^3^. Undiagnosed iron overload can cause hemochromatosis, in which the excess iron stored in the organs causes severe tissue damage. Furthermore, iron overload is commonly seen in industrialized countries due to the widespread consumption of red meat and iron supplements^4^. Etiologically, the multiple organ dysfunctions are linked to the presence of excess free iron that elevates oxidative damage by generating reactive oxygen species (ROS)^5^ and depleting intracellular antioxidant levels^6^. Iron deposition in the hepatic cells significantly increases the risk of fibrosis and cirrhosis, consequently increasing morbidity and mortality^7^. Furthermore, the liberated ROS can cause hepatic inflammation by inducing specific proinflammatory mediators, including nuclear factor kappa B (NF-κB) and tumor necrosis factor-alpha (TNF-α), which contribute to the pathogenesis and development of both acute and chronic liver damage, culminating in cirrhosis^8^. Therefore, iron homeostasis should be preserved by maintaining an adequate iron supply while preventing excess iron accumulation.

The currently used iron-chelating medications, such as deferiprone, deferoxamine, and deferasirox, have several undesirable side effects, including agranulocytosis, hepatic or renal failure, ocular toxicity, ototoxicity, and growth delay^9,10^.

Compared to manufactured medications, herbal remedies are safer and have fewer adverse effects. Furthermore, several plants are abundant in bioactive chemicals with potent pharmacological activities^11,12^. The *Alnus* genus belonging to the family Betulaceae includes approximately 30 trees and climber species. *Alnus incana* (L.) Moench is extensively distributed in the Northern Hemisphere and has been used to treat gastrointestinal and skin ailments^13^. It is also used for gargling during bacterial mouth and throat infections^14^. This species has been reported to contain natural compounds known as diarylheptanoids with different pharmacological activities, including anti-inflammatory, anti-influenza, and hepatoprotective. Although Sajid et al.^15^ and Kim et al.^16^ reported that the hepatoprotective activities of the closely related species, *Alnus nitida*, and *Alnus japonica,* respectively, this activity has not been reported yet in *A. incana* extracts.

Recently, scientists have been increasingly investigating medicinal plants containing abundant natural chelating phytochemicals with high phenolic content and potent antioxidant activity to use them for iron removal in thalassemic patients^17,18^. Therefore, this study investigated the possible hepatoprotective, iron-chelating, antioxidant, anti-inflammatory, and antiapoptotic activities of the total methanol extract and butanol fraction from *Alnus incana* leaves on iron overload-mediated hepatotoxicity in rats. We also identified the bioactive components in both extracts using ultraperformance liquid chromatography-electrospray ionization-quadrupole time-of-flight mass spectrometry (UPLC-ESI-QTOF-MS) analysis.

## Results

### Phytochemical investigation

The phytochemical analysis of the total extract showed the presence of glycosides tannins, phenolic compounds, flavonoids, sterols and/or triterpenes, with the absence of alkaloids, saponins and anthraquinones (Table 1).

**Table 1:** Phytochemical screening of total and butanol extracts from *Alnus incana* leaves.

Chemical constituents	Total extract	Butanol extract
Carbohydrates / Glycosides	+	+
Phenols / Tannins	+	+
Flavonoids	+	+
Anthraquinones	–	–
Alkaloids / Nitrogenous bases	–	–
Saponins	–	–
Sterols/Triterpenes	+	–

+  = present, – = absent.

### In vitro antioxidant activity

**Figure 1:**
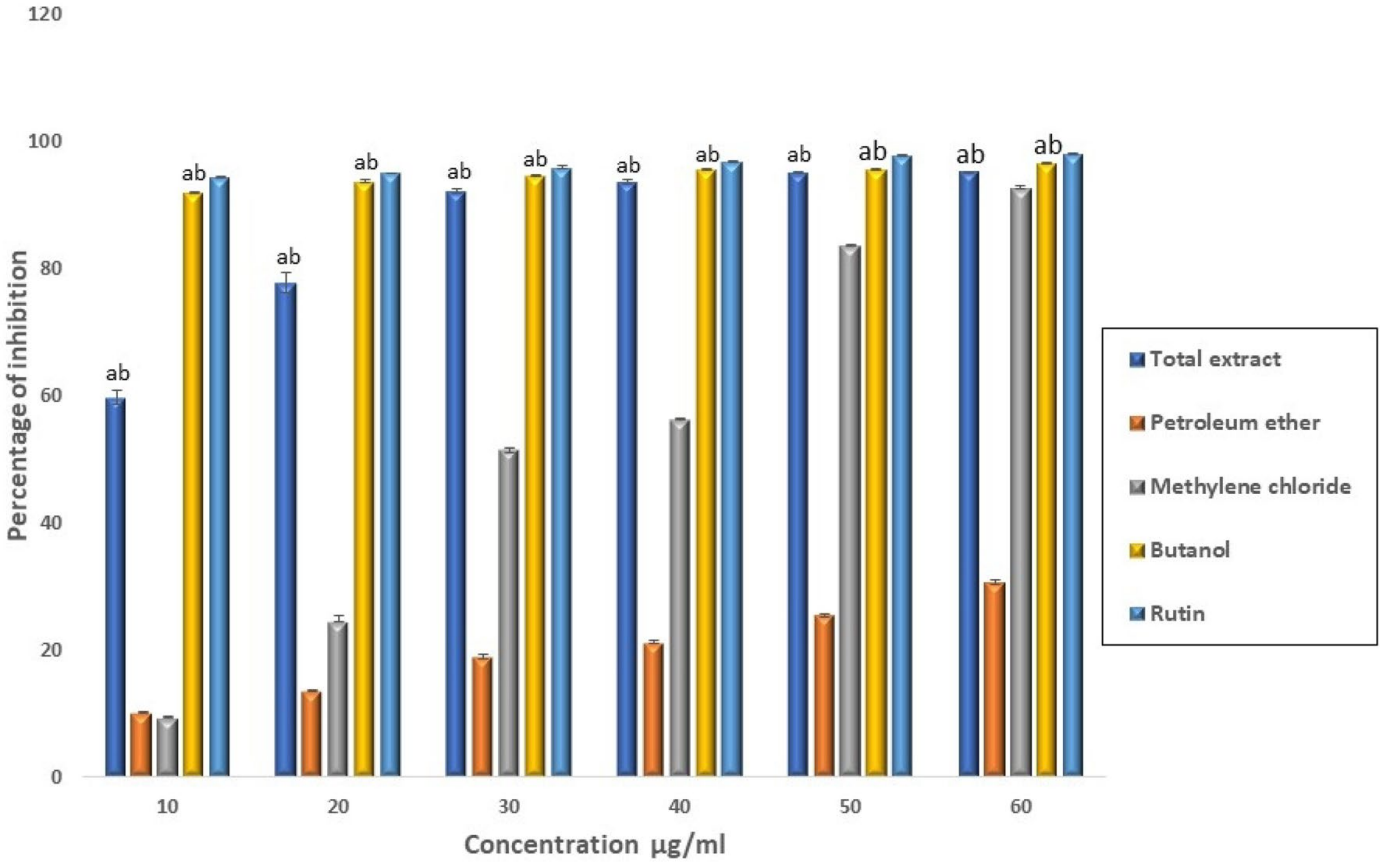
DPPH scavenging activity of different *A. incana* extracts versus standard rutin. Data are expressed as mean ± SEM of three experiments. ^a^Significantly different from petroleum ether extract**,** ^b^Significantly different from methylene chloride extract.

The butanol and total extracts revealed dose-dependent inhibition of 2,2-diphenyl-1-picrylhydrazyl (DPPH) activity with half-maximal inhibitory concentration (IC_50_) values of 0.015 and 7.55 µg/ml, respectively. On the other hand, IC_50_ in the petroleum ether and methylene chloride fractions was 229, and 29.06 µg/ml, respectively. All concentrations of both total extract and butanol fraction significantly inhibited the free radicals compared to methylene chloride and petroleum ether fractions (*P* < 0.05) (Fig. 1). Hence, we evaluated the potential ameliorative effects of total *Alnus incana* extract and butanol fraction against the hepatotoxicity induced by iron overload in male albino rats.

### Total phenolic and flavonoid content

The total phenolic and flavonoid content of the total extract was found to be 125.88 ± 10.5 mg gallic acid equivalent (GAE) /g and, 122.4 ± 11.5 mg catechin equivalent (CE)/g respectively, while that of the butanolic fraction was 245 ± 18.5 GAE/g and 100 ± 9.8 mg CE/g, respectively (Table 2).

**Table 2:** Total phenolic and flavonoid content in total extract and butanol fraction from *Alnus incana* leaves.

Sample	Polyphenols (mg GAE/g)	Total flavonoids (mg CE/g)
Total extract	125.88 ± 10.5	122.4 ± 11.5
Butanol extract	245 ± 18.5	100 ± 9.8

Results are expressed as mean ± SEM (standard error of the mean) of three replicate determinations.

*GAE* Gallic acid equivalent, *CE* Catechin equivalent.

### UPLC-ESI-QTOF/MS analysis of *A. incana* total extract and butanol fraction

Fifty and forty-three polyphenolic compounds were identified in *A. incana* total extract and butanol fraction, respectively, including flavonoids (35, 29), phenolic acids (8,7), stilbenes (1, 1), coumarin (2, 2), and diarylheptanoids (4, 4), respectively (Table 3). The flavonoids comprised aglycones and O- or C-glycosides of flavonols, flavones, isoflavones, flavanones, chalcones, and anthocyanidins. Stilbenes, chalcones anthocyanidins and phenolic acids represented the classes of the highest concentration (800.38%, 247.81%, 247.29%, 242.39%, and 101.49%, respectively) in the butanol fraction as compared to total extract. Furthermore, daidzein-8-C-glucoside, malvidin-3-glucoside and oregonin were the compounds of the highest abundance in butanol fraction as compared to the total extract calculated from the peak area (Table 3).

**Table 3:** Secondary metabolites tentatively identified in *A. incana* total extract and butanol fraction using UPLC-ESI-QTOF-MS analysis.

Peak no.	Total extract			Butanol fraction			MS/MS fragments	Molecular formula	Tentatively identified compounds
	RT min	Precursor m/z	Area	RT min	Precursor m/z	Area			
Flavonoids:									
Flavonoids (Flavonols):									
1	4.49	477.0641	98,501				433, 315	C_22_H_22_O_12_	Isorhamnetin-3-O-glucoside
2	5.40	433.2046	14,608	5.55	433.207	233,262	387, 179	C_20_H_18_O_11_	Quercetin-3-D-xyloside
3	6.51	463.0878	91,518	6.53	463.0909	2,210,855	317, 316, 301, 300, 287	C_21_H_20_O_12_	Myricitrin
4	6.55	463.0881	410,231	6.69	463.0591	98,425	301, 179, 151	C_21_H_20_O_12_	Quercetin-4′-glucoside
5	6.73	507.186	4,012,482	6.73	507.1871	170,264	327, 205	C_23_H_24_O13	Syringetin-3-O-galactoside
6	7.18	417.0915	50,358	7.16	417.127	95,458	349, 286.9	C_20_H_18_O_10_	Kaempferol-3-O-alpha-L-arabinoside
7	7.22	447.0924	1,686,477	7.24	447.0924	11,006,731	301, 300, 271, 255	C_21_H_20_O_11_	Quercitrin
8	7.96	431.099	613,739	8.00	431.0985	1,516,820	385, 284, 255, 227	C_21_H_20_O_10_	Kaempferol-3-O-alpha-L-rhamnoside
9	8.49	317.0536	105,702	8.45	317.0461	55,848	316, 271, 179	C_15_H_10_O_8_	Myricetin
10	8.71	593.1522	8821	8.75	593.1512	237,968	447, 285, 284	C_27_H_30_O_15_	Datiscin
11	9.29	315.1607	2,701,822	9.30	315.1611	6,779,784	–	C_16_H_12_O_7_	Isorhamnetin
12	9.36	593.2583	854,753	9.37	593.2612	1,628,689	461, 299	C_30_H_26_O_13_	Kaempferol-3-O-(6-p-coumaroyl)-glucoside
13	9.43	623.1636	54,370	9.46	623.1586	153,268	577, 283	C_28_H_32_O_16_	Isorhamnetin-3-O-rutinoside
14	9.67	301.0329	112,476	9.52	301.0352	2,658,260	179, 151, 121	C_15_H_10_O_7_	Quercetin
						52.9% @			
Flavonoids (Flavones):									
15	5.43	577.1556	30,985	5.45	577.1563	456,548	431, 413, 311, 293, 269	C_27_H_30_O_14_	Vitexin-2ʺ-O-rhamnoside
16	5.54	431.1157	47,331				341, 311, 283	C_21_H_20_O_10_	Apigenin 8-C-glucoside (Vitexin)
17	6.94	609.2164	20,775	6.89	609.2565	134,287	447	C_27_H_30_O_16_	Luteolin-7, 3′-di-O-glucoside
18	9.21	445.1873	72,390	7.03	445.1868	53,504	269	C_21_H_18_O_11_	Baicalein-7-O-glucuronide (Baicalin)
19	9.14	591.1731	9016	9.02	591.1729	30,070	283, 268	C_28_H_32_O_14_	Acacetin-7-O-rutinoside
20	11.17	299.0565	5,318,264	11.23	299.0566	3,654,774	284, 255, 227	C_16_H_12_O_6_	Diosmetin
21	11.45	577.1389	193,888				269	C_27_H_30_O_14_	Rhoifolin
22	13.85	269.0452	50,535	13.87	269.0464	21,579	225, 151, 117	C_15_H_10_O_5_	Apigenin
23	14.01	269.0459	4,260,195	14.06	269.0454	1,506,480	241, 225, 197, 169	C_15_H_10_O_5_	Baicalein
24	14.18	283.0607	13,331,249	14.41	283.0611	721,984	268, 239, 211	C_16_H_12_O_5_	Acacetin
						20.73%@			
Flavonoids (Flavanones):									
25	8.59	609.2551	434,969	8.63	609.2551	183,399	463, 301	C_28_H_34_O_15_	Hesperetin-7-O-neohesperidoside
26	9.51	301.0303	57,521				286, 177, 151	C_16_H_14_O_6_	Hesperetin
27	10.02	271.0582	408,307	10.16	271.0577	64,839	227, 151, 119	C_15_H_12_O_5_	Naringenin
						27.55%@			
Flavonoids (Isoflavonoids):									
28	7.11	415.1007	136,412	7.12	415.1037	782,590	295, 267	C_21_H_20_O_9_	Daidzein-8-C-glucoside (Puerarin)
29	7.7	267.1241	47,102				223, 195	C_16_H_12_O_4_	Formononetin
30	13.46	253.0501	15,138,962	13.49	253.0502	9,864,519	225, 133	C_15_H_10_O_4_	Daidzein
						69.48%@			
Flavonoids (Chalcones):									
31	7.41	449.0973	100,505	7.35	449.1004	313,904	287, 151	C_21_H_22_O_11_	Okanin-4′-O-glucoside
32	7.93	435.1093	25,414	7.96	435.1093	67,639	389, 273	C_21_H_24_O_10_	Phlorizin
33	10.04	611.2049	28,046				449, 303	C_28_H_36_O_15_	Neohesperidin dihydrochalcone
						247.81%@			
Flavonoids (Anthocyanidins):									
34	8.52	491.116	186,642	8.65	491.1214	1,372,790	476, 329, 313	C_23_H_25_O_12_	Malvidin-3-glucoside (Oenin)
35	8.62	609.1294	434,969	8.571	609.1293	164,395	447, 301	C_27_H_31_O_16_	Delphinidin-3-O-(6ʺ-O-alpha-rhamnopyranosyl-beta-glucopyranoside)
						247.29%@			
Phenolic acids and their derivatives:									
36	1.15	153.0189	384,809	1.23	153.0184	1,254,224	109	C_7_H_6_O_4_	3,4-dihydroxybenzoic acid
37	1.32	167.0348	113,911	1.34	167.0352	223,247	123	C_8_H_8_O_4_	Homogenentisic acid
38	1.92	163.0406	44,141	1.81	163.0407	96,371	119	C_9_H_8_O_3_	3-(4-hydroxyphenyl) prop-2-enoic acid
39	4.2	137.0238	1,508,186	4.26	137.0228	2,515,764	93	C_7_H_6_O_3_	P-hydroxybenzoic acid
40	6.79	167.0340	252,046	6.84	167.0354	1,542,414	152, 108	C_8_H_8_O_4_	5-Methoxysalicylic acid
41	7.38	167.0704	72,380				123, 93	C_8_H_8_O_4_	3-Hydroxymandelic acid
42	9.23	359.1511	9,690,364	9.4	359.1517	19,923,818	341, 179	C_18_H_16_O_8_	Rosmarinic acid
43	15.41	385.1854	232,273	14.49	385.2005	4,253,580	223, 207	C_17_H_22_O_10_	1-O-b-D-glucopyranosyl sinapate
						242.39%@			
Coumarin:									
44	6.93	177.0561	220,525	6.96	177.0553	765,040	135, 121	C_9_H_6_O_4_	6,7-dihydroxycoumarin (Esculetin)
45	14.06	339.1601	810,558	14.93	339.1581	281,364	177	C_15_H_16_O_9_	Esculin
						101.49%			
Stilbene:									
46	8.55	405.1196	46,557	8.60	405.1198	372,634	243, 159	C_20_H_22_O_9_	E-3,4,5′-trihydroxy-3′-glucopyranosyl stilbene (Astringin)
						800.38% @			
Diarylheptanoids:									
47	7.14	477.1761	30,914,226	7.30	477.1771	44,452,316	327, 205, 121	C_24_H_30_O_10_	Oregonin
48	8	461.182	2,471,443	8.03	461.1824	3,595,057	311, 205	C_24_H_30_O_9_	Alnuside A
49	8.14	475.1967	1,545,211	8.17	475.1969	2,318,811	295, 189, 179	C_25_H_32_O_9_	Platyphylloside
50	8.36	493.208	7,704,394	8.34	493.2072	6,102,773	331	C_25_H_34_O_10_	Rubranoside A
						132.44%@			

@ Percentage of each class of identified constituents in butanol fraction relative to total extract calculated from peak area.

The percentage of abundance of each class was calculated by dividing the total peak areas of each class in butanol by total peak areas in total extract multiplied by 100.

% of daidzein-8-C-glucoside, malvidin-3-glucoside and oregonin in butanol fraction as compared to the total extract, calculated from the peak area (573.69%, 735.52%, 143.79% respectively).

The identified flavonoids from *A. incana* total extract and butanol fraction included quercetin, myricetin, baicalin, baicalein, apigenin, naringenin, puerarin, and malvidin-3-glucoside, which were identified from their mass spectra by reviewing library database and available literature (Figure Suppl. 1).

### Acute toxicity study

During the entire experimental period, no signs of acute toxicity and mortality were observed at doses up to 1000 mg/kg body weight (b.w.). Hence, the dose of butanol fraction was selected as 100 mg/kg b.w. for further studies.

### Effect of treatment on serum liver function parameters

Iron-overloaded group revealed a substantial (*P* < 0.05) decrease in the albumin and total protein levels and a marked (*P* < 0.05) rise in the total bilirubin level, aspartate aminotransferase (AST), and alanine aminotransferase (ALT) activities compared to their controls (Table 4). Treatment with both extracts and the reference drug markedly (*P* < 0.05) elevated the albumin and total protein levels and significantly (*P* < 0.05) decreased the total bilirubin level, ALT, and AST activities compared to that of the iron-overloaded rats. Furthermore, the butanol extract showed substantially higher levels of the parameters mentioned above than the reference drug-treated rats.

**Table 4:** Serum albumin, total protein, total bilirubin, ALT, and AST activities among different experimental groups.

	C	Iron overloaded	Iron overloaded + T	Iron overloaded + B	Iron overloaded + D
Albumin g/dl)	4.63 ± 0.087	3.08 ± 0.06^a^	4.14 ± 0.09^ab^	4.43 ± 0.08^bc^	4.03 ± 0.06^ab^
Total protein (g/dl)	7.15 ± 0.09	5.12 ± 0.09^a^	6.21 ± 0.23^ab^	6.91 ± 0.1^bcd^	6.05 ± 0.08^ab^
Total bilirubin (mg/dl)	0.30 ± 0.01	0.83 ± 0.032^a^	0.51 ± 0.016^ab^	0.38 ± 0.011^abcd^	0.52 ± 0.018^ab^
ALT (U/L)	23.60 ± 0.44	60.2 ± 2.39^a^	30.52 ± 1.12^abc^	24.39 ± 0.41^bcd^	37.49 ± 0.67^ab^
AST (U/L)	34.2 ± 0.76	89.4 ± 2.36^a^	39.4 ± 1.58^bc^	36.2 ± 1.13^bc^	49.73 ± 1.05^ab^

Results are expressed as mean ± SEM (standard error of the mean) (n = 6 rats/group).

C: Control group, Iron overloaded: Iron overloaded group, Iron overloaded + T: Iron overloaded + total extract-treated group, Iron overloaded + B: Iron overloaded + butanol fraction-treated group, Iron overloaded + D: Iron overloaded + deferoxamine-treated group.

*ALT* Alanine aminotransferase, *AST* Aspartate aminotransferase.

^a^Significantly different from control, ^b^Significantly different from Iron overloaded group, ^c^Significantly different from Iron overloaded + D group, ^d^Significantly different from Iron overloaded + T group.

### Effect of treatment on both serum and hepatic iron content

**Figure 2:**
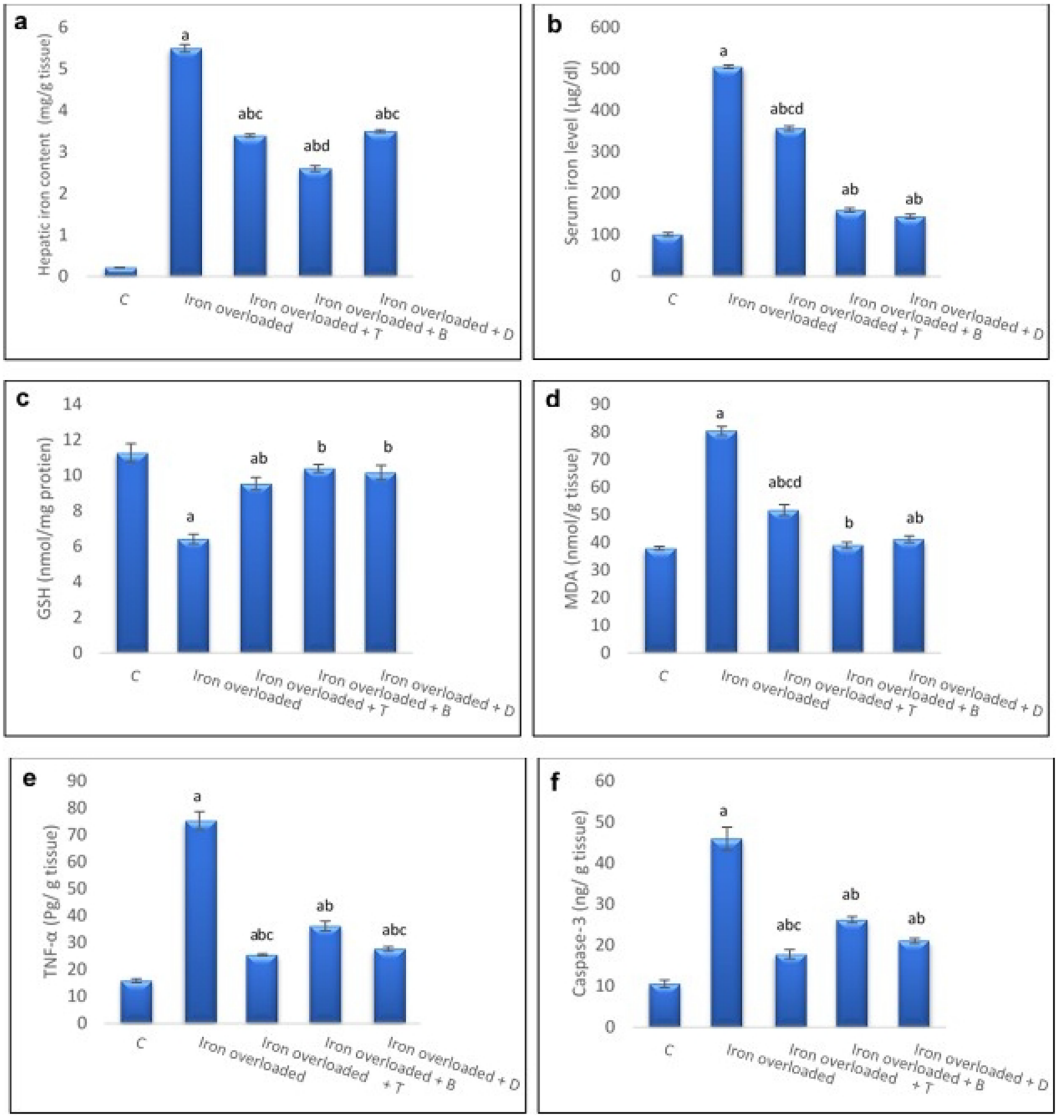
Hepatic iron content (**a**), serum iron level (**b**), GSH (**c**), MDA (**d**), TNF-α (**e**), and Caspase-3 (**f**) levels among different experimental groups. C: Control group, Iron overloaded: Iron overloaded group, Iron overloaded + T: Iron overloaded + total extract-treated group, Iron overloaded + B: Iron overloaded + butanol fraction-treated group, Iron overloaded + D: Iron overloaded + deferoxamine-treated group. Results are expressed as Mean ± S.E.M (n = 6 rats/group). ^a^significantly different from control, ^b^significantly different from Iron overloaded group, ^c^significantly different from Iron overloaded + B group, ^d^significantly different from Iron overloaded + D group.

The iron-overloaded group revealed considerably higher serum and hepatic iron content than the control (*P* < 0.05). However, the administration of the total extract, butanol fraction, and deferoxamine to the iron-overloaded rats significantly decreased these parameters compared to the overloaded group (*P* < 0.05). The serum iron level in the butanol extract-treated rats was substantially lower than those in the total extract-treated rats (*P* < 0.05) without any significant difference (*P* > 0.05) from the levels in the reference drug-treated rats. The butanol extract treatment significantly decreased the hepatic iron content compared to those treated with the total extract and the reference drug (*P* < 0.05) (Fig. 2a and b).

### Effect of treatment on oxidative stress biomarkers

Iron administration substantially (*P* < 0.05) elevated the hepatic malondialdehyde (MDA) content and concomitantly reduced the hepatic reduced glutathione (GSH) content compared to their normal controls. While treatment with both extracts and the reference drug significantly reduced the MDA content and increased the GSH content compared to the iron-treated rats (*P* < 0.05). The reduction in the MDA level was markedly higher in the butanol extract than in the total extract. Contrarily, treatment with butanol extract did not show a significant difference (*P* > 0.05) in the GSH level compared to the control group (Fig. 2c and d).

### Effect of treatment on hepatic TNF-α level, caspase-3 activity

Iron overload significantly increased the hepatic TNF-α level and caspase-3 activity compared to their control values (*P* < 0.05) (Figs. 2e and f). Conversely, the administration of the total extract, butanol fraction, and the reference drug to iron-overloaded animals substantially reduced these parameters compared to the overloaded rats (*P* < 0.05). The rats treated with the total extract displayed significantly (*P* < 0.05) lower TNF-α level and caspase-3 activity than the butanol fraction-treated rats.

## Histopathology

### Light microscopy

**Figure 3:**
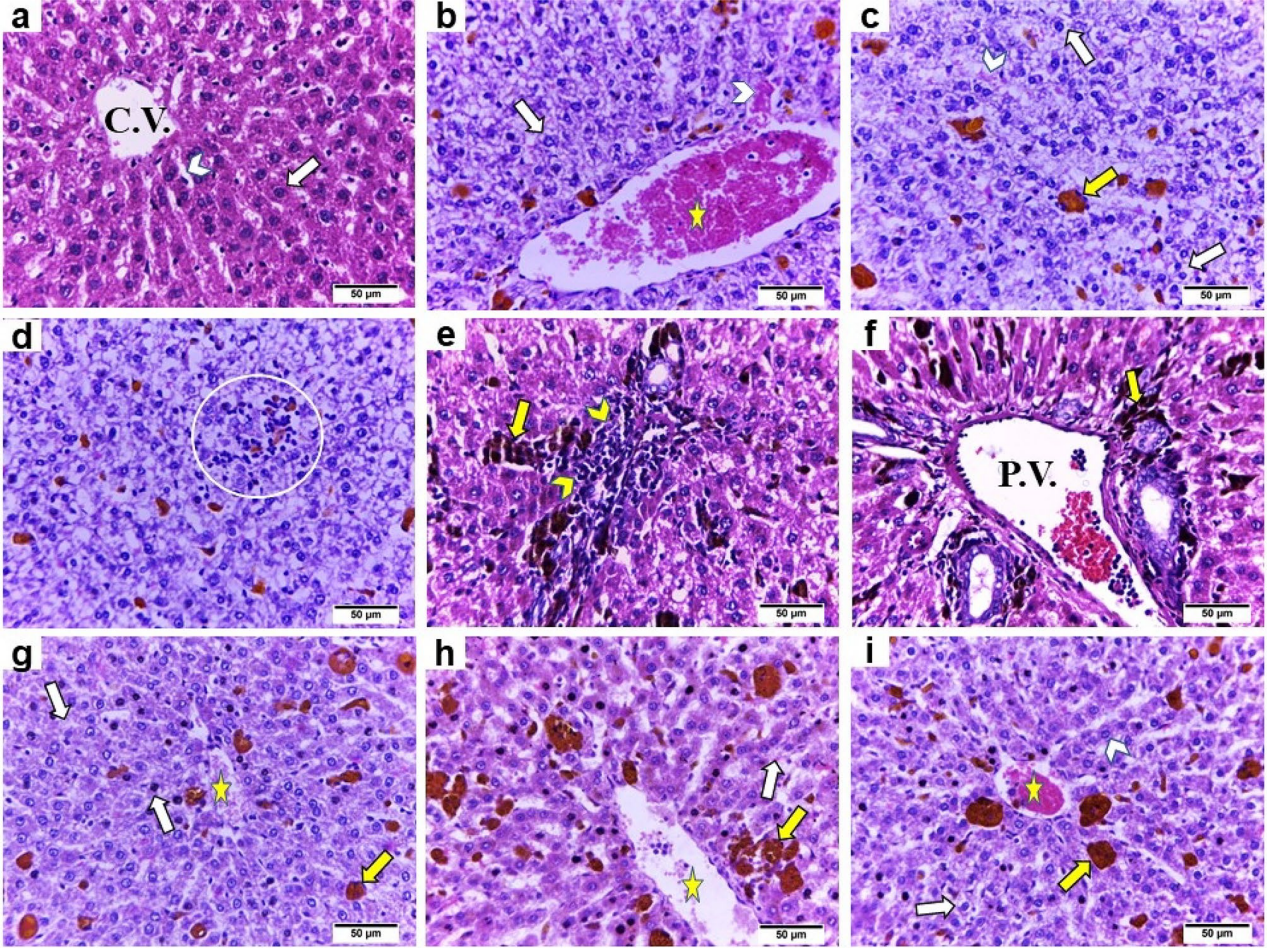
Representative photomicrographs of liver tissue sections of different experimental groups (H&E, × 400): (**a**) Control group displaying normal histological architecture of hepatic tissue with central vein (C.V.) and regular hepatic plates (arrow) separated by hepatic sinusoids (arrowhead) (**b**:**f**) Iron-overloaded group showing severe hepatic injury, including dilatation and congestion of central vein (star) and hepatic sinusoids (white arrowhead), disarranged hepatic cords, hepatocellular degeneration with cytoplasmic vacuolization (white arrow), yellow–brown iron deposits (yellow arrow), inflammatory cells infiltration between hepatocytes (circle) and in the portal area (yellow arrowhead) along with dilatation and congestion of portal vein (P.V.). (**g**:**i**) Cotreated groups (III, IV, and V) demonstrating marked attenuation in the iron overload-induced hepatic injury accompanied by a decrease in the iron accumulation (yellow arrow). (**g**) Iron-overloaded group cotreated with total extract of *Alnus incana* exhibiting remarkable improvement in the histological structure with mild dilatation and congestion of the central vein (star) and mild hepatocellular degeneration (white arrow). (**h**) Iron-overloaded group cotreated with butanol extract of *Alnus incana* revealing an apparently normal hepatic parenchyma with only mild dilatation and congestion of central vein (star), and mild hepatocellular degeneration (white arrow). (**i**) Iron-overloaded group cotreated with deferoxamine markedly restored the hepatic parenchyma with straightly arranged hepatic cords (arrowhead), moderate dilatation, and congestion of central vein (star) along with mild hepatocellular degeneration  and cytoplasmic vacuolization (white arrow).

The liver sections from the control rats displayed normal histological architecture. The liver consisted of the central vein with regular hepatic plates separated by hepatic sinusoids. Each hepatic plate contained large polygonal hepatocytes with central, rounded, and vesicular nuclei (Fig. 3a). However, the iron-overloaded rats exhibited severe hepatic injury, including dilatation and congestion of the central vein and hepatic sinusoids, disarranged hepatic cords, hepatocellular degeneration with cytoplasmic vacuolization, yellow–brown iron deposits, inflammatory cells infiltration between hepatocytes and in the portal area along with dilatation and congestion of the portal vein (Fig. 3b–f). Conversely, the cotreated groups revealed marked attenuation in the iron overload-induced hepatic injury. The iron-overloaded rats cotreated with total extract of *Alnus incana* exhibited remarkable improvement in the histological structure with mild dilatation and congestion of the central vein and mild hepatocellular degeneration (Fig. 3g). The liver sections from group IV exhibited an apparently normal hepatic parenchyma with only mild dilatation and congestion of central vein and mild hepatocellular degeneration (Fig. 3h). Contrastingly, coadministration with deferoxamine markedly restored the hepatic parenchyma with straightly arranged hepatic cords, moderate dilatation, and congestion of central vein along with mild hepatocellular degeneration and cytoplasmic vacuolization (Fig. 3i).

Our results (Table 5) showed that the highest histopathological lesion score was observed in the iron-overloaded group (II). Contrastingly, the cotreated groups (III, IV, and V) revealed a marked reduction in all microscopic lesion scores.

**Table 5:** Pathological lesion scoring in the liver sections of different experimental groups.

Histopathological lesions/groups	C	Iron overloaded	Iron overloaded + T	Iron overloaded + B	Iron overloaded + D
Dilatation and congestion of central vein	–	++++	+	+	+ +
Hepatocellular degeneration	–	+++	+	+	+
Inflammatory cells infiltration					
a-between hepatocytes	–	+	–	–	–
b-portal area	–	++	–	+	+
Dilatation and congestion of portal vein	–	+++	+	++	++

(–) normal histological structure, (+) mild, (++) moderate, (+++) severe damage, and (++++) more severe damage (n = 6 rats/ group). C: Control group, Iron overloaded: Iron overloaded group, Iron overloaded + T: Iron overloaded + total extract-treated group, Iron overloaded + B: Iron overloaded + butanol fraction-treated group, Iron overloaded + D: Iron overloaded + deferoxamine-treated group.

### Prussian blue staining

**Figure 4:**
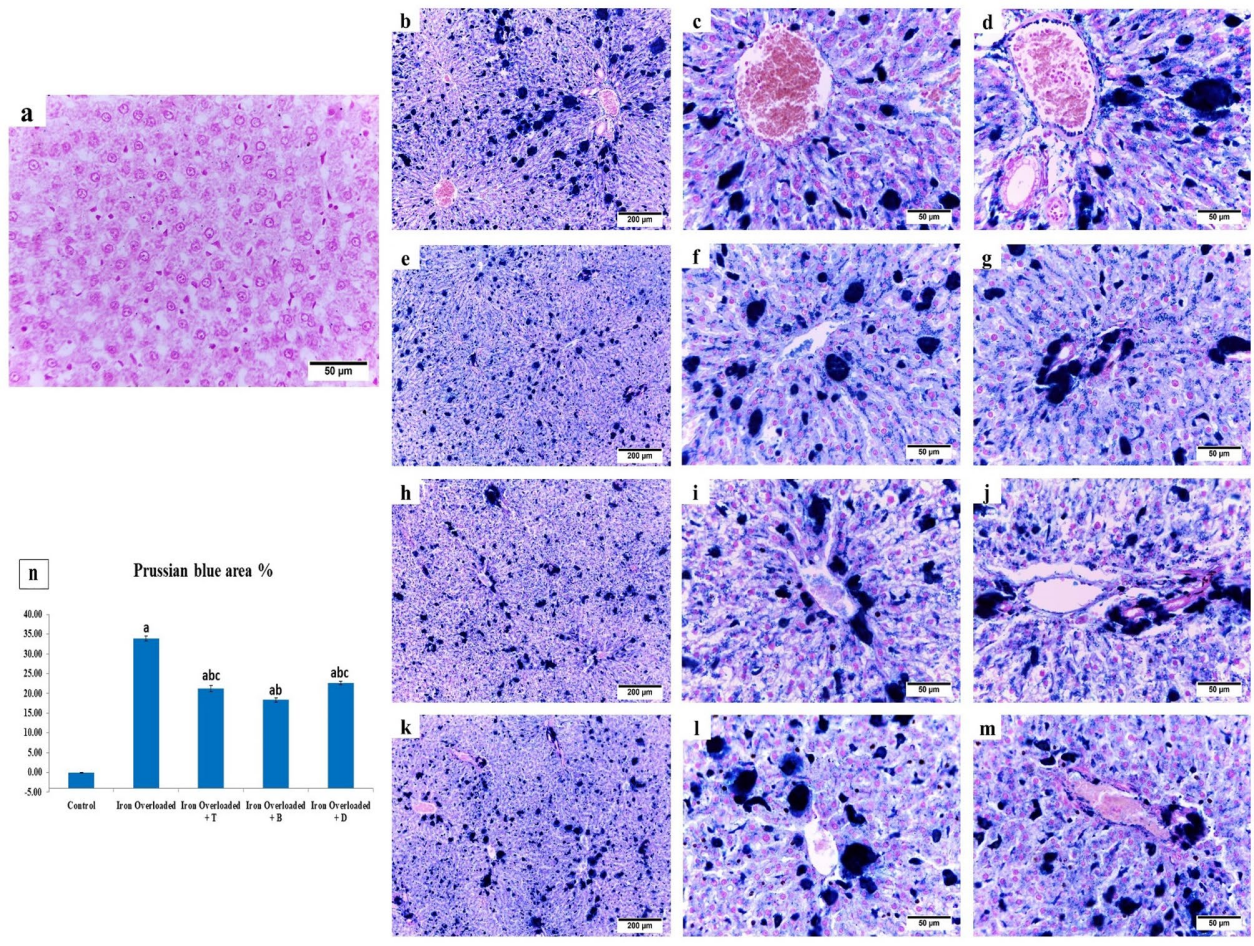
Photomicrographs representing the distribution (**a**–**m**) and quantification (**n**) of Prussian blue stained iron deposits in the hepatic tissue sections of different groups. (**a**) Control group, (**b**:**d**) Iron overloaded group, (**e**:**g**) Iron overloaded group co-treated with total extract of *Alnus incana*, (**h**:**j**) Iron overloaded group co-treated with butanolic extract and **(k:m) **Iron overloaded group co-treated with deferoxamine. (**a**,**b**,**e**,**h**,**k**: × 100) (**c**,**f**,**i**,**l**: High magnification of the central area, × 400) (**d**,**g**,**j**,**m**: High magnification of the portal area, × 400). (**n**) Quantification of Prussian blue stain positive area % in the hepatic tissue of rats. Results are expressed as Mean ± S.E.M. ^a^significantly different from control, ^b^significantly different from Iron overloaded group, ^c^significantly different from Iron overloaded + B group, ^d^significantly different from Iron overloaded + D group.

Prussian staining was performed to visualize the distribution of iron deposits in the hepatic tissue sections and confirm the variation between the different experimental groups. The liver sections from the control group displayed no iron deposits (Fig. 4a). Conversely, the blue pigment represented the iron deposits in the cytoplasm of the hepatocytes, interstitium, and portal area in the other treated groups (Fig. 4b–m). The highest hepatic iron accumulation was observed in the iron-overloaded rats (Fig. 4b–d) with a mean area % (33.92 ± 0.57, Fig. 4n) compared to other co-treated groups (Fig. 4e–m). Whereas treatment with total (Fig. 4e–g) and butanolic (Fig. 4h–j) extract as well as deferoxamine (Fig. 4k–m) markedly (*P* < *0.05*) attenuated hepatic iron accumulation with mean area % (21.25 ± 0.78, 18.33 ± 0.59, and 22.59 ± 0.47, respectively, Fig. 4n).

### Immunohistochemistry

#### Caspase 3

**Figure 5:**
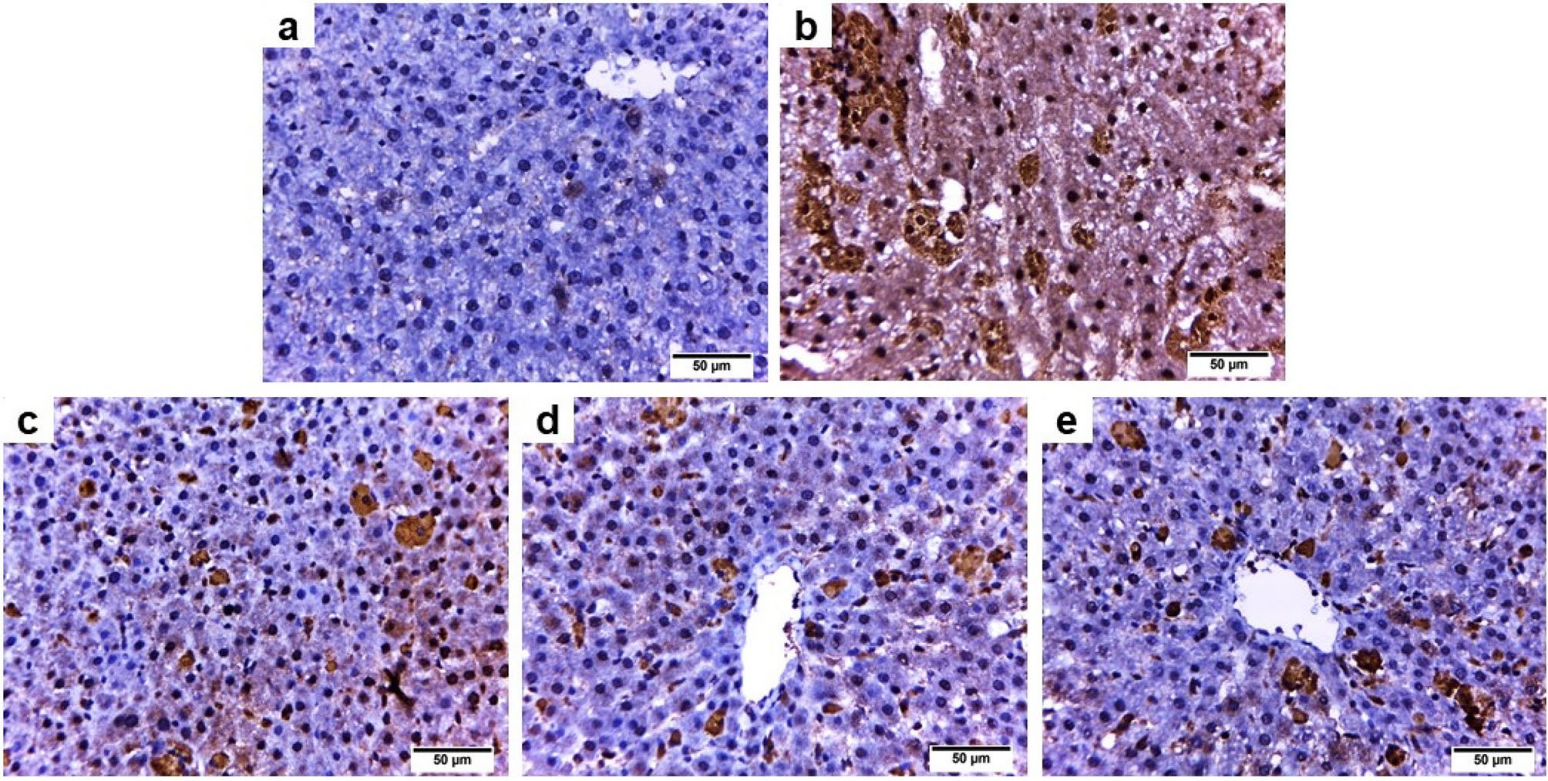
Photomicrographs of caspase 3—stained liver sections from rats in the different experimental groups (× 400). (**a**) Control group, (**b**) Iron overloaded group, (**c**) Iron overloaded group co-treated with total extract of *Alnus incana*, (**d**) Iron overloaded group co-treated with butanol, and (**e**) Iron overloaded group co-treated with deferoxamine.

The liver sections from the iron-overloaded group (Fig. 5b) showed stronger positive immunoreactivity for caspase 3 than the control group (Fig. 5a). Whereas the iron-overloaded group cotreated with the total extract or butanol fraction or deferoxamine revealed moderate caspase 3 immunoreactivity compared to group II (Fig. 5c–e, respectively).

#### NF-κB

**Figure 6:**
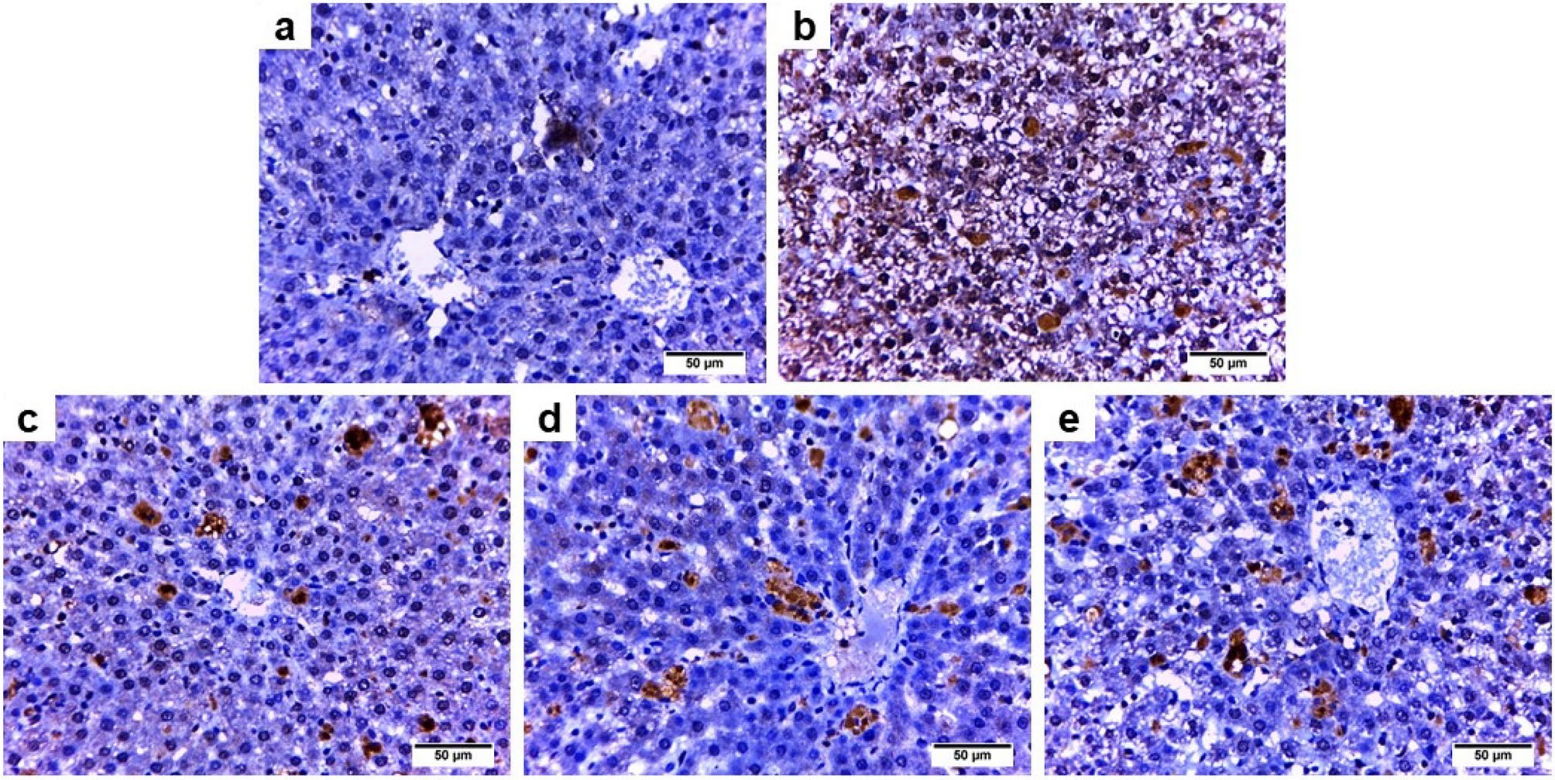
Photomicrographs of NFκB—stained liver sections from rats in the different experimental groups (× 400). (**a**) Control group, (**b**) Iron overloaded group, (**c**) Iron overloaded group co-treated with total extract of *Alnus incana*, (**d**) Iron overloaded group co-treated with butanol, and (**e**) Iron overloaded group co-treated with deferoxamine.

The liver sections from the iron-overloaded group (Fig. 6b) exhibited higher positive immunoreactivity for NF-κB than the control group (Fig. 6a). Conversely, the iron-overloaded group cotreated with total extract (group III) or butanol fraction (group IV), or deferoxamine (group V) revealed milder NF-κB immunoreactivity than group II (Fig. 6c–e, respectively).

#### Correlation studies

The current results revealed that the hepatic iron level significantly correlated with hepatic TNF-α, caspase-3, MDA, serum iron level, ALT, AST, and total bilirubin level, where r = 0.813**, 0.833**, 0.798**, 0.823**, 0.845**, 0.813**, and 0.910** respectively. On the other hand, a significant negative correlation was observed between the hepatic iron content and hepatic GSH content, serum albumin, and total serum protein where r = − 0.798** − 0.87** and − 0.869** respectively (**P < 0.01). Similarly, the serum iron level significantly correlated with hepatic TNF-α, caspase-3, MDA, ALT, AST, and total bilirubin level, where r = 0.791**, 0.750**, 0.933**, 0.792**, 0.786**, and 0.865** respectively. Also, serum iron level was negatively correlated with hepatic GSH, serum albumin, and total protein where r = − 0.798**, − 0.830**, and − 0.779**, (**P < 0.01).

## Discussion

Iron, an essential microelement, has several vital roles in living organisms^19,20^. However, iron overload, occurring due to poisoning, diseases, and pathological conditions, can induce multiorgan dysfunction, including liver damage^21^. The currently used iron-chelators such as deferiprone, deferoxamine, and deferasirox, cause several undesirable side effects, including agranulocytosis, neutropenia, gastrointestinal disturbances, and hepatic or renal failure^9,10^. These disadvantages of the current iron chelating agents highlight the innovation for alternative and safe pharmacological interventions. Ebrahimzadeh et al.^18^ showed that herbal extracts with high phenolic content could act as chelators or adjuvants to treat iron overload. Therefore, here, we evaluated the antioxidant, iron chelating, anti-inflammatory, and antiapoptotic potentials of methanol extract from *Alnus incana* leaves and its butanol fraction against iron overload-induced hepatotoxicity.

Our results indicated that excess iron intake increased serum and hepatic iron levels, accompanied by significant alterations in liver function and lipid peroxidation. Subsequently, this resulted in deficient antioxidant defense mechanisms and apoptosis of the hepatic tissues. These findings align with Badria et al.^22^ and Al-Basher^23^.

Based on our results, iron-overloaded rats revealed significantly elevated serum ALT and AST enzyme activities and total bilirubin levels but lower serum albumin and total protein, consistent with Al-Basher^23^. Furthermore, the hepatic iron content significantly correlated with hepatic serum ALT, AST, and total bilirubin level, where r = 0.845**, 0.813**, 0.910**, respectively (**P < 0.01). Whereas the hepatic iron content was negatively correlated with serum albumin, and total serum protein where r =  − 0.87** and − 0.869**, respectively (**P < 0.01). These results were accompanied by a significant increase in the mean area % (33.92 ± 0.57) of hepatic iron accumulation as well as severe histopathological alterations in the form of dilatation, and congestion of central vein and hepatic sinusoids, disarranged hepatic cords, hepatocellular degeneration with cytoplasmic vacuolization, and inflammatory cells infiltration. These findings are consistent with Jahanshahi et al.^24^ and Wang et al.^25^. The substantial elevation in liver function indicates liver injury, which subsequently affects hepatocyte’s transport function, and leakage, resulting in the release of ALT and AST^26,27^. Furthermore, these histopathological alterations might affect hepatic functions and induce hypoalbuminemia and hypoproteinemia. Moreover, excessive iron content causes significant hepatocyte dysfunction, leading to the failure of normal uptake, conjugation, excretion, and subsequent rise in total bilirubin level^28^.

However, administration of both extracts markedly reduced the levels of AST, ALT activities, and total bilirubin and increased the albumin and total protein levels compared to the iron-overloaded rats. In the butanol extract-treated group, the parameters were restored to their normal ranges, accompanied by a marked reduction in the mean area % of the hepatic iron deposits with significantly improved hepatic parenchyma structure. These findings suggest the potent antioxidant effect of both extracts, as evidenced by the measurement of free radicals scavenging activity (Fig. 1). These antioxidant effects could be due to the presence of flavonoids and polyphenols in these extracts, which can stabilize and maintain the integrity of the liver cell membrane, stimulate hepatocyte regeneration, attenuate, and repair the damaged tissue, and subsequently enhance the enzymatic activity of liver and synthesis of hepatocellular proteins^29,30^. Furthermore, butanol administration significantly reduced the hepatic iron content compared to those treated with the total extract and the reference drug, suggesting that it has better iron chelation activity than total extract in the iron-overloaded rats.

The phytochemical profiles of *A. incana* total extract and butanol fraction were identified using UPLC-ESI-QTOF-MS analysis. The data showed that the total extract and butanol fraction were rich in various polyphenols and diarylheptanoids.

The highest iron-chelating activity of the butanol fraction could be attributed to the greater abundance of daidzein-8-C-glucoside, malvidin-3-glucoside, and oregonin in this fraction than the total extract (Table 3). It has been reported that the aforementioned compounds exhibited iron-chelating activity via different mechanisms of action^31–42^. Additionally, flavonoids are the major identified class of constituents in *A.incana* extracts, and can chelate metals more efficiently than other polyphenols, suggesting that they can be used to potentially treat iron overload. According to Wang et al.^42^, flavonoids combat the accumulation of iron through three main mechanisms: (1) reducing iron saturation indirectly via multiple proteins and pathways, such as ferritin and hepcidin; (2) chelating iron to decrease iron accumulation, which directly decreasing iron saturation via binding sites, such as the 6, 7-dihydroxy structure, the B-ring catechol, and the 2, 3-double bond. Baicalin, baicalein, and quercetin have also been reported to exhibit significant iron-chelating activity; (3) oxidation resistance to diminish oxidative damage from iron overload by reacting with superoxide anion radicals to prevent free radicals’ initiation, suppressing the Fenton reaction to prevent hydroxyl radical generation, and reacting with lipid peroxidation groups to prevent lipid peroxidation. This effect may be due to higher quantities of polyphenols in the butanol extract than in the total extract (Table 2). Polyphenols exert their antioxidant activities through different mechanisms including free radical scavenging potentials and iron binding. In general, transition metals involving iron promote the production of oxygen-free radicals, reduce peroxide, and interact with superoxide anions, resulting in oxidative stress. It has been found that polyphenols possess iron-binding abilities; these activities are primarily due to the presence of groups like catechol and galloyl. Besides, some investigations revealed that the 6,7-dihydroxy structure, B-ring catechol, the galloyl groups, the 2,3-double bond, and the 3- and 5-hydroxylic groups in co-existence with the 4-keto group are linked to chelation abilities, consequently, they are worthy of consideration as iron-binding sites^42^.

In this study, excess hepatic iron content induced a substantial (*P* ˂ 0.05) elevation in the hepatic MDA content along with a marked (*P* ˂ 0.05) decrease in the hepatic GSH level in the iron-overloaded groups compared to the control ones. These findings indicate oxidative stress via enhancement of lipid peroxidation, excess free radical formation, and deficient antioxidant mechanism, which subsequently leads to hepatic dysfunction and leakage of hepatic enzymes^43,44^. Papanikolaou and Pantopoulos^45^ showed that excess iron creates free radicals, which induce damage to cellular macromolecules and potentiate cell death. Our results suggest a significant correlation between the hepatic iron content and MDA level (r = 0.798**) and a negative correlation between the hepatic iron content and GSH content (r =  − 0.798**). Conversely, treatment with total extract and butanol fraction attenuated the induced oxidative stress as evidenced by a marked (*P* ˂ 0.05) elevation in GSH content and a significant (*P* ˂ 0.05) decline in MDA level compared to the iron-overloaded rats. These results might be attributed to the antioxidants found in both extracts. The butanol extract revealed greater antioxidant effects than the total extract. As this fraction contains higher polyphenol content (245 mg GAE/g) than the total extract (126 mg GAE/g, Table 2), the antioxidant activity correlates to the polyphenol concentration^46^. Natural polyphenols are potent antioxidants because they can scavenge free radicals, chelate metals, convert primary oxidation products to non-radical molecules, break the chain to prevent the continuous loss of hydrogen from substrates, and regulate antioxidant enzymes^47^.

Our study confirmed that iron overload-induced apoptosis manifested by potent caspase 3 immunoreactivity with markedly (*P* ˂ 0.05) elevated liver caspase 3 activity. This substantially correlated with the hepatic iron content. Previous research demonstrated that excess iron-induced oxidative stress which in turn caused damage to the inner mitochondrial membrane and initiated the opening of the mitochondrial pores. As a result, adenosine triphosphate (ATP) is depleted, and cytochrome c is released into cytosol that subsequently activates caspase 3 and induces cell death^8,48^. The activation of caspase 3, a crucial apoptosis mediator, causes DNA fragmentation and apoptotic chromatin condensation in the cells^49^. Therefore, excess iron promotes liver cell necrosis or apoptosis either alone or in combination with oxidative stress^48^. Furthermore, our results demonstrated that inflammation is another possible mechanism of iron overload-induced hepatotoxicity as the iron overload group revealed significantly (*P* ˂ 0.05) higher hepatic TNF-α activity accompanied by strong NF-κB immunohistochemical expression. Handa et al.^50^ reported that excessive iron supplementation induces oxidative stress in the liver cells, initiating both inflammatory and immune mediators as well as hepatocellular ballooning injury, which results in the prognosis of nonalcoholic steatohepatitis. TNF-α, a multifunctional cytokine, is crucial for various physiological and pathological mechanisms, including inflammation, apoptosis, and necrosis^51^. Furthermore, TNF-α activates NF-κB signaling pathways by activating toll-like receptors, resulting in increased immunohistochemical expression of NF-κB^52,53^. The activation of NF-κB can initiate and regulate the expression of a series of inflammatory cytokines implicated in inflammation^54,55^. Additionally, NF-κB is regulated by the excessive generation of ROS and many cytokines, including Interleukin-1 beta^56^. Our results demonstrated that both extracts have potent anti-inflammatory and antiapoptotic activities manifested by mild NF-κB immunoreactivity, moderate caspase 3 immune expression with substantially (*P* ˂ 0.05) lower liver TNF-α and caspase 3 activities than the iron-overloaded group. This might be attributed to the potent antioxidant capability of these extracts, along with their ability to chelate and inhibit hepatic iron contents. Moreover, the total extract exhibited higher anti-inflammatory and antiapoptotic activities than the butanol extract, possibly due to the synergistic effects of several polyphenolic compounds, including phenolic acids, flavonoids, stilbenes, coumarins, lignins, and tannins, which exhibit anti-inflammatory and antiapoptotic activities^57–62^. Besides, other classes, namely terpenoids and diarylheptanoids, displaying similar activities^63–66^ were also detected in this extract (Tables 1 and 3).

Our results indicate that deferoxamine treatment significantly decreases the serum and hepatic iron contents and liver biomarkers (AST and ALT), consistent with Mansi et al.^20^, Jahanshahi et al.^24^, and Wang et al.^25^. Moreover, this treatment significantly reduced elevated levels of MDA, TNF-α, and caspase-3 and increased GSH contents, consistent with Wang et al.^25^. Furthermore, deferoxamine significantly attenuated the hepatic iron deposition, improved hepatic damage, and decreased caspase-3 and NF-κB immunoreactivity induced by the iron overload, which aligns with the observations by Jahanshahi et al.^24^ and Wang et al.^25^. These results are attributed to its potent chelating activity. Heli et al.^67^ reported that iron chelators could diminish oxidative stress by eliminating excess iron from the target tissues.

## Conclusions

Our study demonstrated that both total and butanol extracts from *Alnus incana* could markedly chelate excessive iron and ameliorate the iron overload-induced hepatotoxicity in rats by reducing serum and hepatic iron contents, liver biomarker activities, diminishing oxidative stress, inhibiting inflammation and apoptosis, resulting in significantly higher serum albumin, total protein, and total bilirubin content, and improved endogenous antioxidant activity. Furthermore, they can alleviate the histopathological and histochemical alterations induced by iron overload due to their phytochemical contents. These findings suggest that both extracts might represent a novel treatment for iron overload-induced hepatoxicity and other pathological conditions characterized by hepatic iron overload, including thalassemia and sickle-cell anemia.

## Materials and methods

### Drugs and chemicals

All the chemicals were of high analytical grade and obtained from Sigma-Aldrich (St. Louis, MO, USA). Deferoxamine was purchased from Novartis Pharma AG (Basel, Switzerland).

### Plant material

*Alnus incana* (L.) Moench leaves were collected with permission from Al Zoharia research garden following institutional, national, and international guidelines. Dr. Mamdouh Shokry, a botanist at Al Zoharia Research Garden in Cairo, Egypt, validated and authenticated the plant material. A voucher specimen was deposited at the herbarium of Pharmacognosy Lab, Faculty of Pharmacy (Girls), Al-Azhar University under the number (AR-2016).

### Extract preparation

The air-dried plant material (1 kg) was extracted by thoroughly macerating it with 100% methanol (3×, 3 L) and filtered through filter paper (Whatman no. 1). The collected filtrate was dried under vacuum (45 °C) by rotary evaporator to obtain the total extract. Then, 350 g of the total extract had been suspended in distilled water, then successively fractionated using gradient polarity solvents, including petroleum ether, methylene chloride, and n-butanol. The fractions were filtered and evaporated by rotary evaporator using a suitable temperature to obtain the petroleum ether (60 g), methylene chloride (220 g), and n-butanol (12 g) fractions.

### Phytochemical screening

We performed qualitative tests to detect the different phytochemical classes in the total and butanol extracts from *Alnus incana* leaves, according to^68^.

### Determination of total phenolic and flavonoid content

The total phenolic content was determined using the Folin–Ciocalteu procedure^69^. The total flavonoid content was quantified using State Pharmacopeia of USSR^70^.

### UPLC-ESI-QTOF-MS analysis of *A. incana*

UPLC-ESI-QTOF-MS is a sophisticated and sensitive technique to identify secondary plant metabolites qualitatively and quantitatively. A negative ESI technique was applied to detect the various phytoconstituents in the *A. incana* total extract and butanol fraction.

The *A. incana* total extract and butanol fraction (100 mg) was dissolved in 2 mL of methanol: acetonitrile: water (1:1:2) by vortexing for 2 min and ultrasonicating for 10 min. After centrifuging for 10 min at 10,000 rpm, the solution was diluted to reach a final concentration of 2.5 µg/µL. Then, 10 µL was used for injection in the negative mode. The molecules were separated using an Axion AC system (Kyoto, Japan) linked to an autosampler system with an In-Line filter disk precolumn (0.5 µm × 3 mm, Phenomenex, USA) and an Xbridge C18 (3.5 µm × 2.1 mm × 50 mm) column (Waters Corporation, Milford, MA, USA) maintained at 40 °C and flow rate of 300 μL/min. Gradient elution was applied using the mobile phase, which consists of 5 mM ammonium formate in 1% methanol (pH = 8) and 100% acetonitrile. Triple TOF™ 5600 + system outfitted with a Duo Spray™ source operating in the ESI mode (AB SCIEX, Concord, Canada) was used for MS analysis. Following each scan, the top 15 intense ions were selected for obtaining the MS/MS fragmentation spectra. The target analytes were recognized by relating the LC/MS data with the reference database (ReSpect negative, 1573 records) and previously published compounds^71^.

### In vitro antioxidant activity

The free radical scavenging potential of the extracts had been evaluated by the method described by^72^. IC_50_ for all extracts was determined by probit analysis^73^.

### Animals

Sixty male Wistar rats (210–230 g) were obtained from the National Organization for Drug Control and Research's animal house. They were kept under standard environmental conditions including a regular light/dark cycle (12/12 h), 25 °C ± 1 °C room temperature, and 50% ± 4% relative humidity. After acclimatizing for one week under a natural light cycle with adequate ventilation, they were fed a standard pellet diet and had unrestricted access to water.

### Acute oral toxicity study

Thirty rats were equally assigned to five groups (6 rats/group). All groups were orally administered with increasing doses (125, 250, 500, and 1000 mg/kg b.w.) of the butanol fraction. The group administered with the vehicle alone served as the normal control. After an hour, the rats were observed individually for any abnormal behavioral alterations, such as drowsiness, restlessness, writhing, convulsions, and other symptoms of toxicity and mortality. Then, they were monitored intermittently every 24 h for any acute toxicity symptoms for 14 days^74^.

### Ethics approval and consent to participate

All experimental protocols were approved by the Institutional Animal Care and Use Committee (IACUC) of the Faculty of Veterinary Medicine, Cairo University (Approval No.: Vet Cu 2009 2022484) and conformed to the National Institutes of Health guidelines. All methods were carried out in accordance with ARRIVE guidelines.

## Experimental design

### Experimental animal grouping

Thirty rats were equally assigned to five groups (6 rats/group) as follows:

1. *Control group* rats were maintained on distilled water.
2. *Iron-overloaded group* rats received uniformly six intramuscular injections of 12.5 mg iron dextran/100 g b.w. for 30 days. Meanwhile, rats were administered 60 mg ferrous sulfate /kg b.w. using a gastric tube once daily.
3. *Iron-overloaded* + *total extract of Alnus incana group* Iron-overloaded rats orally received the methanolic extract (200 mg/kg b.w.) according to Sajid et al.^15^ using a gastric tube once daily for one month.
4. *Iron-overloaded* + *butanol fraction group* Iron-overloaded rats orally received butanol fraction (100 mg/kg b.w.) using a gastric tube once daily for one month.
5. *Iron-overloaded* + *reference drug (deferoxamine) group* Iron-overloaded rats intraperitoneally injected with deferoxamine (50 mg/kg b.w.) according to Mirzaei et al.^75^ once daily for a month.

### Sample collection

After two months, blood samples were collected from all groups by retro-orbital plexus puncture, kept for 15 min, and centrifuged for 15 min to separate the serum that was used for assessing the biochemical parameters. After that, the rats were euthanized by cervical dislocation and their livers were either stored for biochemical assay or fixed in 10% neutral-buffered formalin (NBF) for further histopathological investigation.

### Estimation of biochemical parameters

Serum albumin, total protein, total bilirubin, AST, and ALT activities were evaluated according to commercial kits purchased from Biodiagnostics, Cairo, Egypt.

### Estimation of iron-chelating activity

The serum iron level was estimated using a commercial kit purchased from Spectrum Diagnostics, Cairo, Egypt. Specimens of liver tissue were homogenized in cold phosphate-buffered saline, then the hepatic iron content was determined in the homogenates as described by Wootton^76^.

### Estimation of oxidative stress biomarkers

The hepatic homogenates were used to estimate MDA (a lipid peroxidation biomarker), GSH, and total protein concentration based on the methods described by Ohkawa et al.^77^, Ellman^78^, and Bradford^79^, respectively**.**

### Determination of hepatic caspase-3 activity and TNF-α level

We assayed the caspase-3 activity and TNF-α level in the hepatic homogenate using ELISA kits purchased from Cusabio, Germany.

## Histopathological studies

### Light microscopy

The liver tissue specimens were fixed in 10% NBF, processed in alcohol and xylene, embedded in paraffin, sliced into 4 µm-thick sections with a manual microtome, and stained with hematoxylin and eosin (H&E), as previously reported by Bancroft and Gamble^80^. We detected iron deposition and distribution using Prussian blue staining according to Sheehan and Hrapchak^81^. The stained sections were examined and photographed using an Olympus fixed camera.

The hepatic lesions scoring was blindly evaluated in all groups (6 rats/group), and the severity of the pathological lesions was measured as follows: none (−, normal structure), mild changes (+, < 25%), moderate changes (++, 25–50%), severe changes (+++, 51–75%), and very severe changes (++++, > 75%)^44^.

### Quantitative analysis for Prussian-blue-stained samples

For image analysis, five different Prussian blue-stained sections (×100) from each group were examined and quantified the area percentage (area %) of Prussian-blue-stained iron deposits represented by the blue pigmentation using the ImageJ program.

### Immunohistochemical detection of caspase 3 and NF-κB

Liver samples from all experimental groups were subjected to immunohistochemistry (IHC) using caspase 3 and NF-κB antibodies. The paraffin sections were fixed on adhesive slides, rehydrated, subjected to antigen retrieval by heating, then washed and incubated with primary polyclonal antibodies against caspase 3/CPP32 (active form, Diagnostic BioSystems) and NFκB-p65 (Elabscience Biotechnology) at a dilution of 1:100 overnight followed by washing and incubation with horse radish peroxidase for 2 h. After two washes, a diaminobenzidine kit was used to develop the immunostaining, and the brown color represented positive immunoreactivity.

### Statistical analysis

The obtained results were presented as mean ± standard error of the mean (SEM). One-way ANOVA was used in the statistical analysis, which was followed by the Tukey multiple comparison test using SPSS version 21.0. *P* values < 0.05 were considered statistically significant. The correlation coefficient was estimated using linear regression according to Abdel-Daim et al.^82^.

### Supplementary Information

Supplementary Information.

## Data Availability

All data generated or analyzed during this study are included in this published article.
