# Author Correction: The effect of *Alnus incana* (L.) Moench extracts in ameliorating iron overload-induced hepatotoxicity in male albino rats

**DOI:** 10.1038/s41598-023-40311-5

**Published:** 2023-08-22

**Authors:** Fatma Abo-Elghiet, Shaza A. Mohamed, Noha A. E. Yasin, Abeer Temraz, Walid Hamdy El-Tantawy, Samah Fathy Ahmed

**Affiliations:** 1 Pharmacognosy and Medicinal Plants Department, Faculty of Pharmacy for Girls, Al Azhar University, Cairo, Egypt; 2 Cytology and Histology Department, Faculty of Veterinary Medicine, Cairo University, Giza, Egypt; 3 National Organization for Drug Control and Research, Dokki, Cairo, Egypt

Correction to: *Scientific Reports* 10.1038/s41598-023-34480-6, published online 11 May 2023

The original version of this Article contained errors, where the listed classes of plant constituents did not match the data presented in Tables 1 and 3.

As a result, in the Results section, under the subheading ‘Phytochemical investigation’,

“The phytochemical analysis of the total extract showed the presence of tannins, phenolic compounds, flavonoids, alkaloids, saponins, coumarins, terpenoids, anthraquinones, and β-cyanins (Table 1).”

now reads:

“The phytochemical analysis of the total extract showed the presence of glycosides tannins, phenolic compounds, flavonoids, sterols and/or triterpenes, with the absence of alkaloids, saponins and anthraquinones (Table 1).”

In addition, in the Discussion section,

“Besides, other classes, namely saponins, terpenoids, anthraquinones, and diarylheptanoids, displaying similar activities^63,64,65,66^ were also detected in this extract (Table 1).”

now reads:

“Besides, other classes, namely terpenoids and diarylheptanoids, displaying similar activities^63,64,65,66^ were also detected in this extract (Tables 1 and 3).”

The original Article has been corrected.

