# Supplementary material for: The effect of Alnus incana (L.) Moench extracts in ameliorating iron overload-induced hepatotoxicity in male albino rats
Source: Sci Rep. 2023 May 11;13:7635. doi: 10.1038/s41598-023-34480-6 (PMC10175300; doi:10.1038/s41598-023-34480-6)
Supplement: Supplementary file 1 — Supplementary Information. [file 41598_2023_34480_MOESM1_ESM.docx]

**Supplementary Information**

**The effect of *Alnus incana* (L.) Moench extracts in ameliorating iron overload-induced hepatotoxicity in male albino rats**

***Fatma*** ***Abo-Elghiet^1^, Shaza A. Mohamed^1^, Noha A. E. Yasin^2*^, Abeer Temraz^1^, Walid Hamdy El-Tantawy^3^, Samah Fathy Ahmed^3^***

^1^Pharmacognosy and medicinal plants department, Faculty of Pharmacy for Girls*,* Al Azhar University.

^2^Cytology and Histology Department, Faculty of Veterinary Medicine, Cairo University, Giza, Egypt.

^3^National Organization for Drug Control and Research, Dokki, Cairo, Egypt

***LC/MS analysis of A. incana total extract and butanol fraction***

The identified flavonoids from *A.* *incana* total extract and butanol fraction included quercetin, myricetin, baicalin, baicalein, apigenin, naringenin, puerarin, and malvidin-3-glucoside, which were identified from their mass spectra by reviewing library database and available literature as follow:

Compound 9 exhibited a molecular ion peak [M–H] ^−^ at m/z 317 corresponding to the molecular formula C_15_H_10_O_8_ and fragment ions X at m/z 316 [M–2H] ^−^, [X–H–CO–H_2_O] ^−^ at m/z 271 and [1,2A−] at m/z 179. Based on previous studies, this compound was identified as myricetin aglycone (**Figure S1a)** **[1]**. Additionally, compound 14 displayed a [M–H] ^−^ at m/z 301 corresponding to C_15_H_1OO7_ and the daughter ions at *m/z* 179 for [^1,2^A^~~-~~^] and at *m/z* 151 for [^1,3^A^−^]. This compound was tentatively identified as quercetin by reviewing the database and reported literature (**Figure S1b) [1]**.

Among the identified flavones from *A. incabuna* total extract and butanol fraction compound 18, which revealed a [M – H] ^−^ at m/z 445 corresponding to C_21_H_18_O_11_, the base peak was corresponding due to the aglycone at m/z 269 [M – H – 176]^-^ due to the loss of glucuronide moiety. By reviewing the library database and available literature, this compound was assigned to be baicalin, **Figure S1c** **[2]**.

Furthermore, compound 23 demonstrated a [M – H] ^−^ at m/z 269 matching C_15_H_10_O_5_. The ion fragment at m/z 241 resulted from the loss of CO from the 4-position, whereas the ion at m/z 225 resulted from the loss of CO_2_ from the 1-O and 4-CO positions. The ions at m/z 197 and 169 were produced by the sequential loss of CO and CO2 from the ion at m/z 225. based on provided data and the library database it was identified as baicalein **(Figure S1d)** **[2]**.

Compound 22 showed a [M − H] ^−^ ion at m/z 269 matching the molecular formula C_15_H_10_O_5_ and [M − H – CO2] ^−^ at m/z 225 due to the neutral loss of 44 Da (CO_2_) of the C ring. The ions at m/z 151 and m/z 117 typically correspond to the retro-Diels–Alder (RDA) cleavage between the chemical bonds 1 and 3 of the C ring. Accordingly, it was identified as apigenin by comparing the provided data with the library database **(Figure S1e) [3,4].**

Compound 27 exhibited a [M − H] ^−^ ion at m/z 271 corresponding to C_15_H_12_O_5_ and showed the same fragmentation pattern as compound 22 (apigenin aglycone) with the exception of the increase of 2 Da, indicating 2H, in the molecular ion peak proving the presence of flavanone isomer of apigenin. This compound was recognized as naringenin based on the available literature and library database **(Figure S1f) [3].**

From the identified isoflavones from *A. incana*, compound 28 showed a [M – H] ^−^ at m/z 415, corresponding to C_21_H_20_O_9_. The fragment ion [M-H-120]^–^ at m/z 295 is characteristic for the C-glycosides. It was recognized as daidzein-8-C-glucoside, also known as puerarin, based on the pattern of fragmentation that matches the available literature and the reported database, **(Figure S1g) [5].**

Compound 34 revealed a [M – 2H] ^−^ at m/z 491, corresponding to C_23_H_25_O_12_, which further showed a neutral loss of 15 Da (CH_3_) yielding the fragment ion at m/z 476 for [M − 2H – CH3] ^−^. The fragment ion at m/z 329 [M - 2H – 162 (glucose moiety)]^-^ corresponding to the aglycone part, the loss of an additional 42 Da yielded fragment ion at 287 m/z [M - 2H – glucose - CH_2_O]^-^. Accordingly, it was identified as malvidin-3-glucoside based on the available literature and the reported data **(Figure S1h) [6].**

Compound 47 showed a [M – H] ^−^ at m/z 477, which corresponds to the molecular formula C_24_H_30_O_10_. At m/z 327, a base peak ion was formed, indicating a neutral loss of 150 Da related to the removal of xylose moiety. The characteristic fragment ion at m/z 205 indicates the removal of C_7_H_6_O_2_. It was recognized as oregonin from its mass data which was in accordance with previously published data **(Figure S1i) [7]**. Oregonin is one of the well-known diarylheptanoids reported from *A. incana* **[8].**


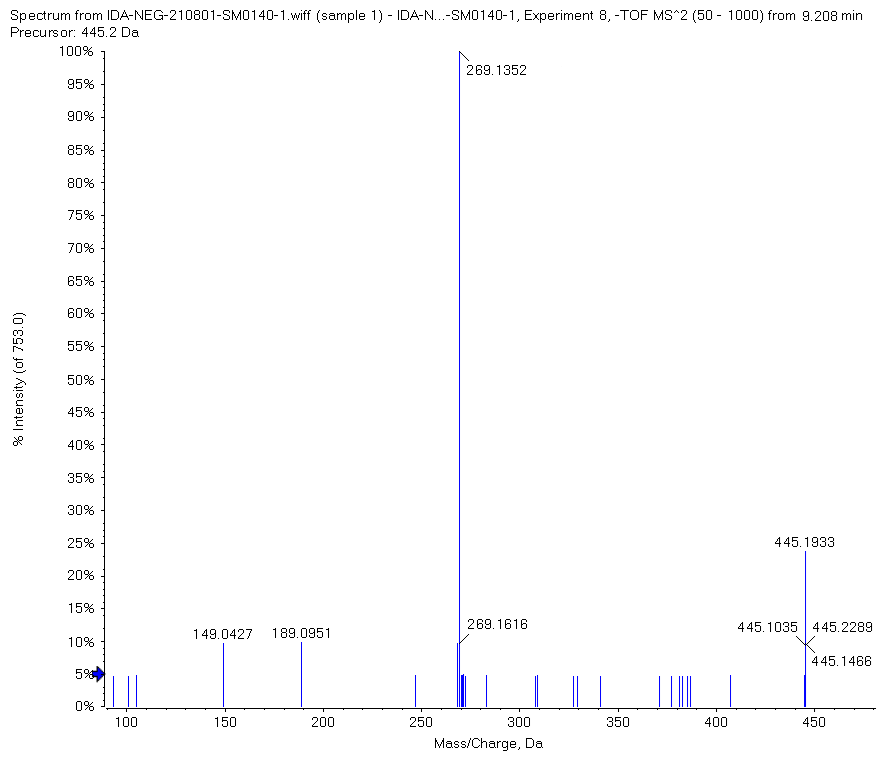

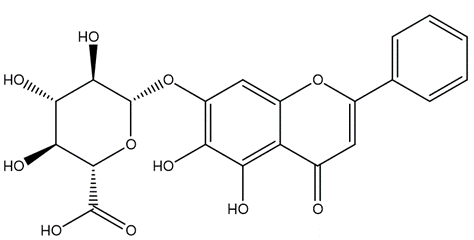


**c**


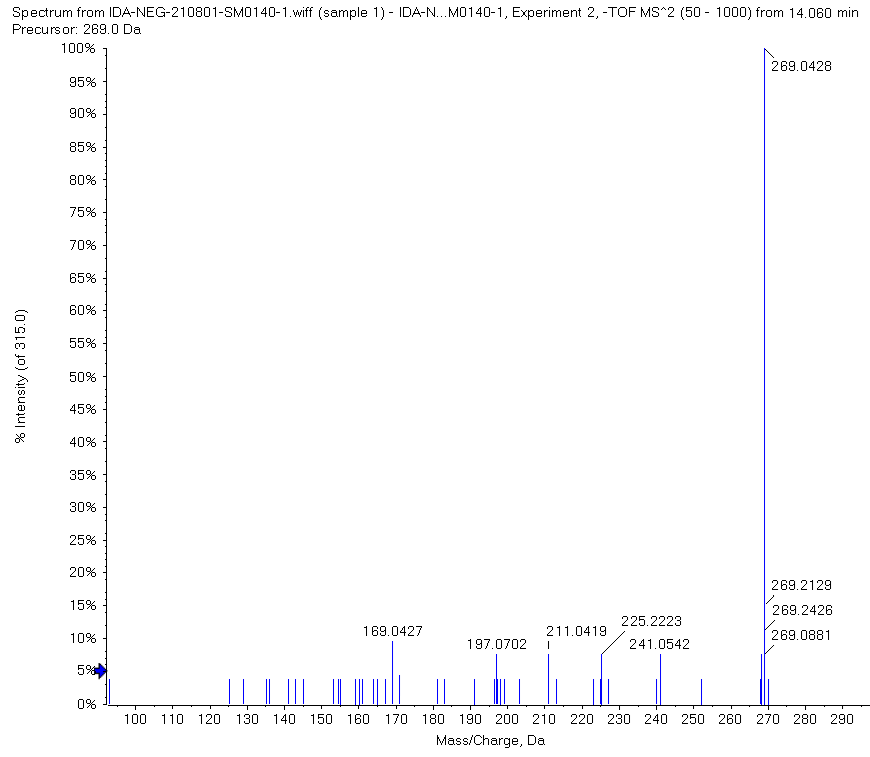

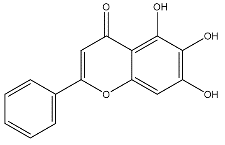


**d**


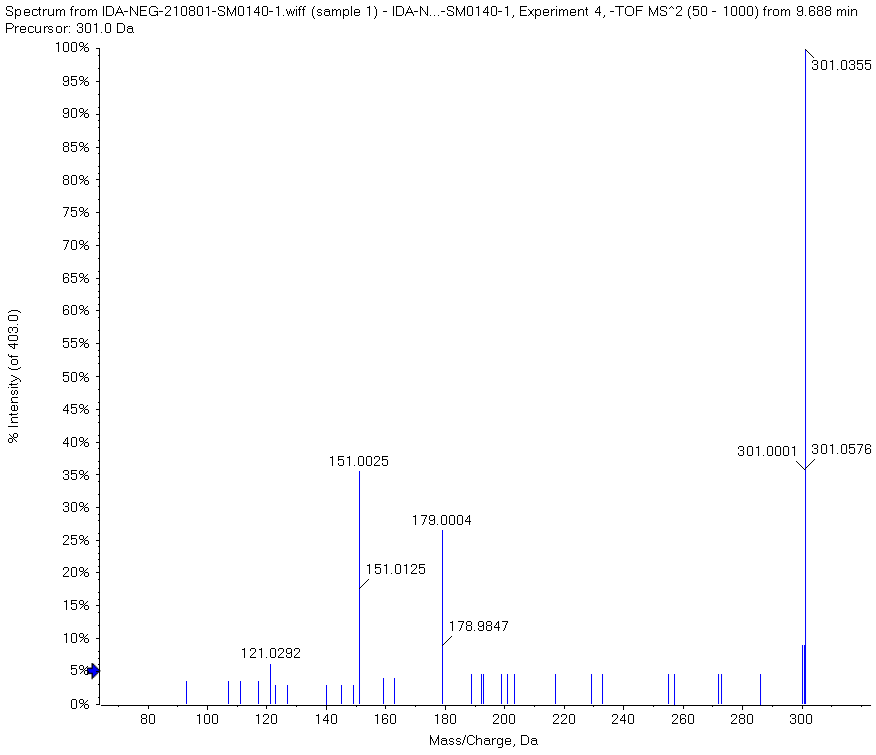

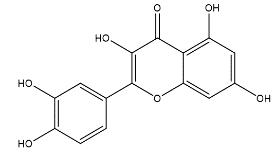


**b**


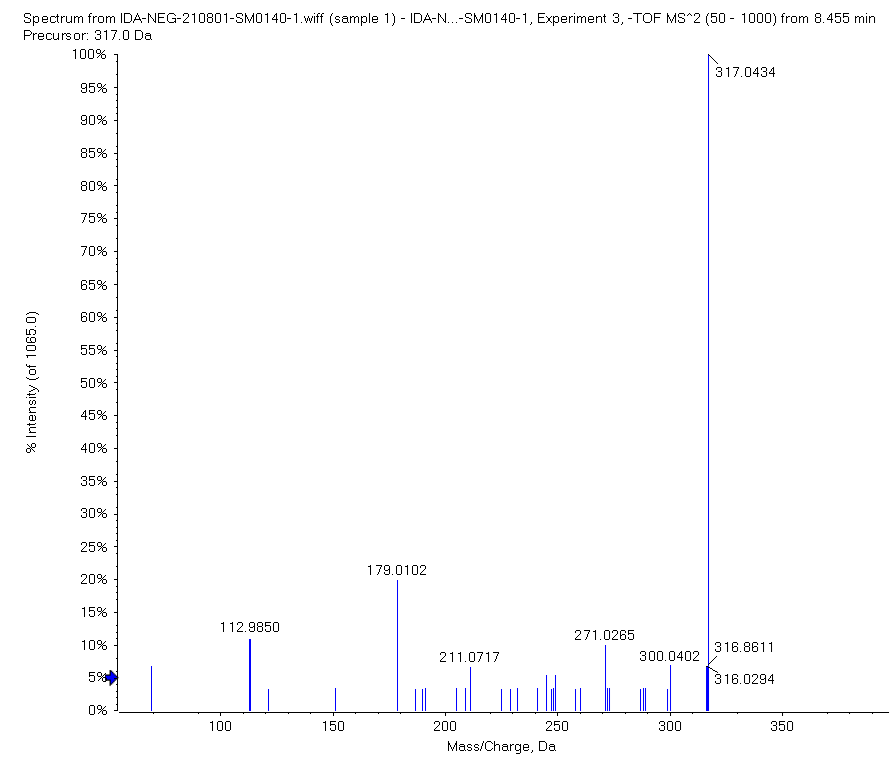

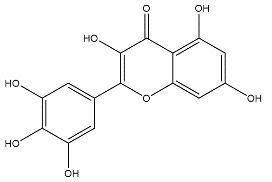


**a**


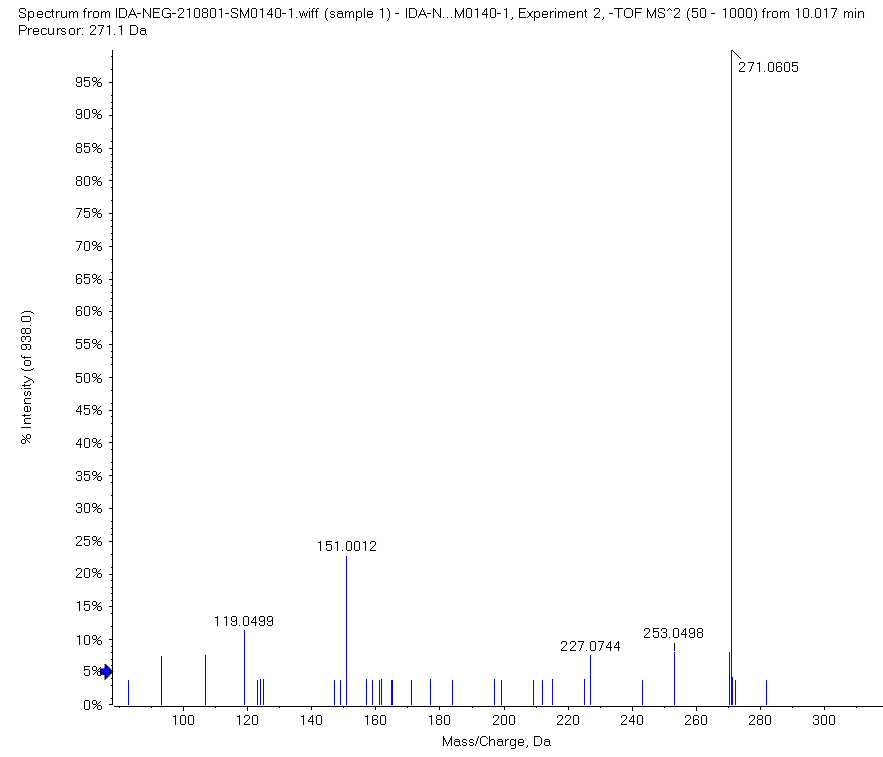

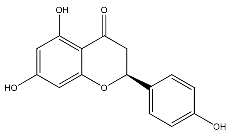


**f**


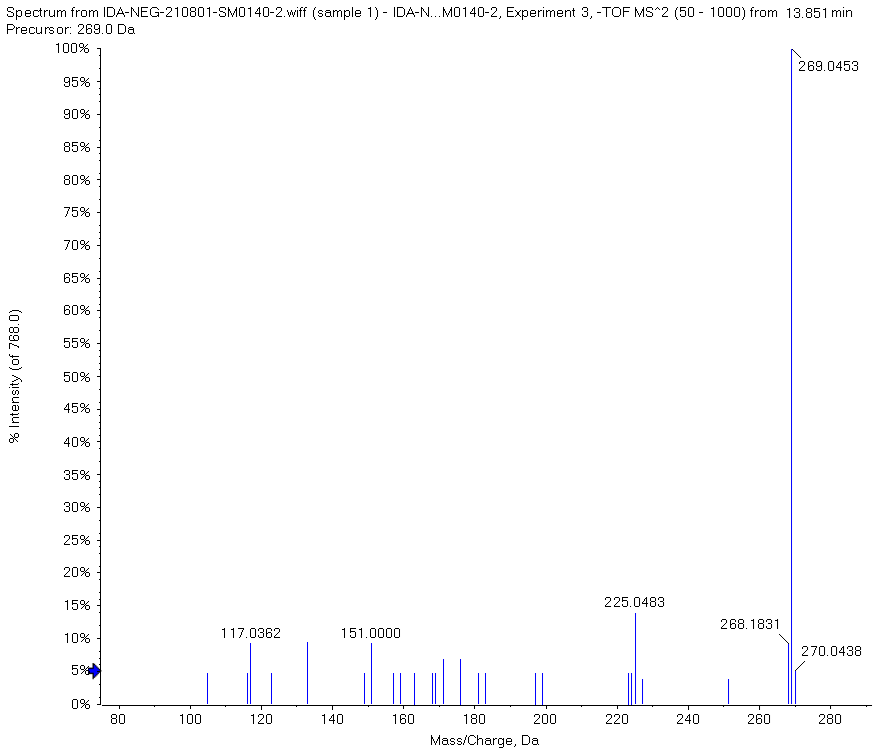

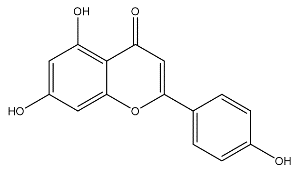


**e**


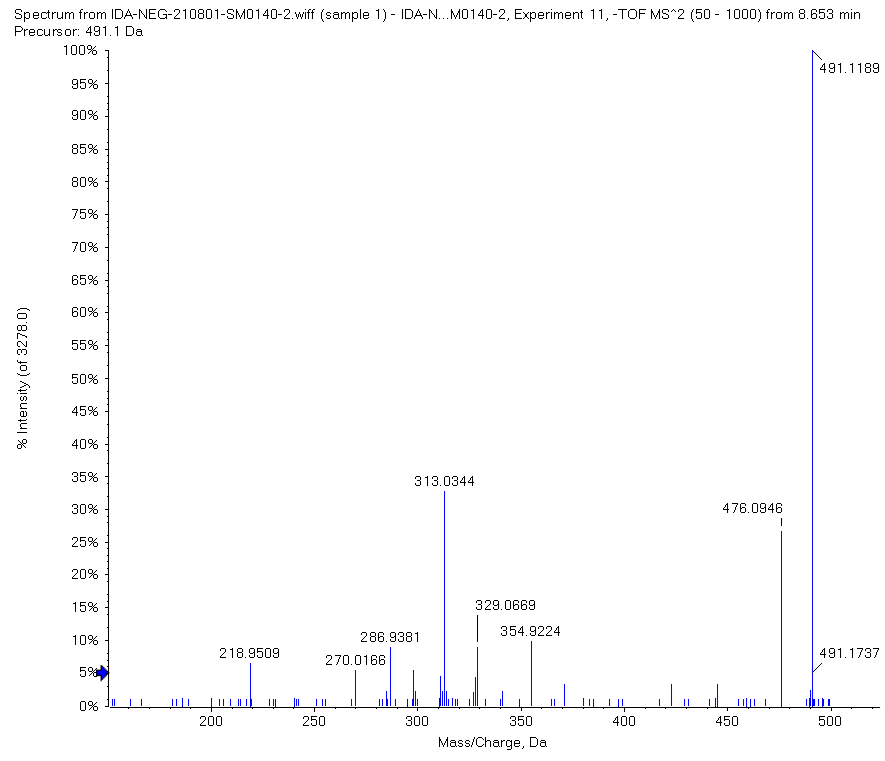

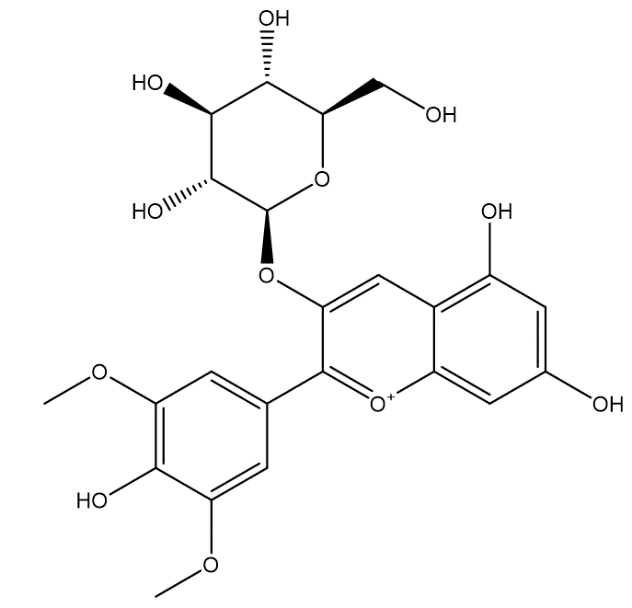


**h**


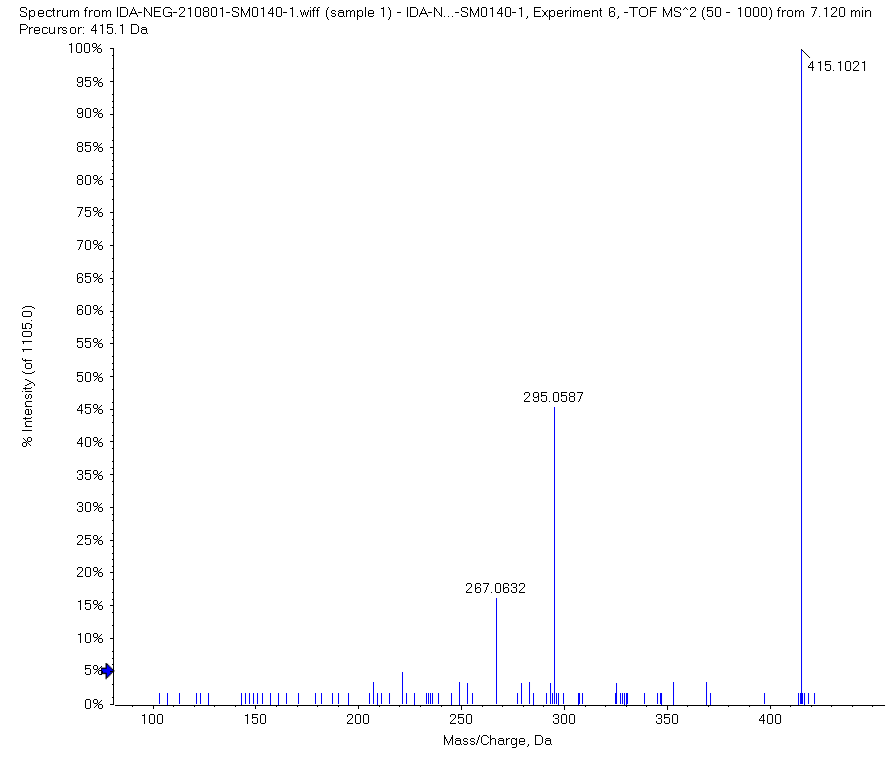

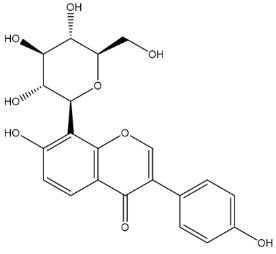


**g**


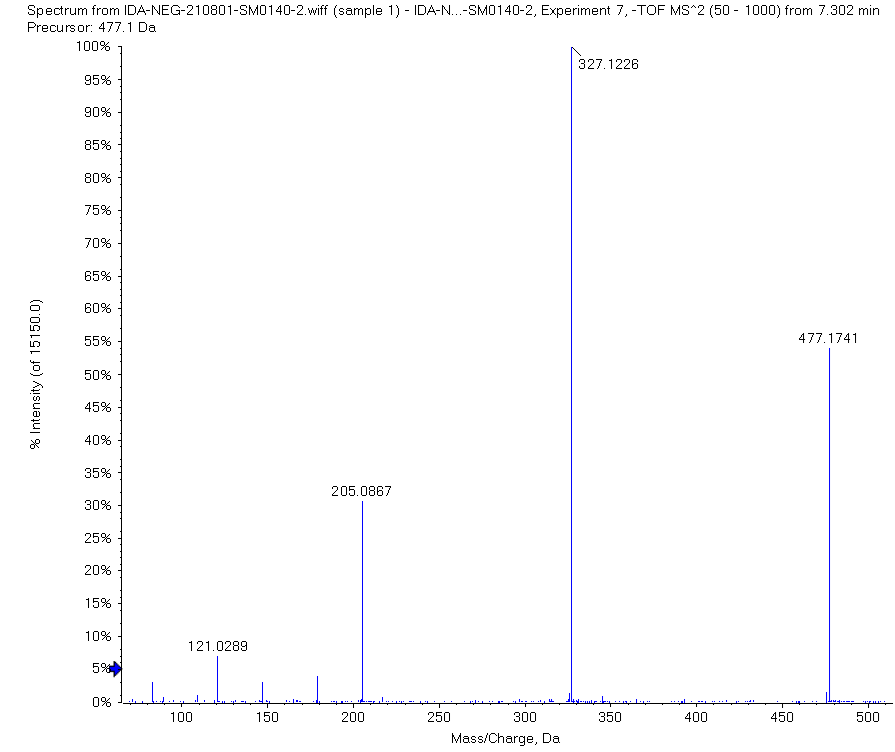

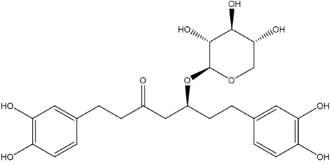


**i**

**Figure. S1. Mass from *A. incana* total and butanol extracts (a) myricetin, (b) quercetin, (c) baicalin, (d) baicalein, (e) apigenin, (f) naringenin, (g) puerarin, (h) malvidin-3 spectra of some identified compounds -glucoside, and (i) oregonin.**

**References:**

1. Saldanha LL, Vilegas W, Dokkedal AL. Characterization of flavonoids and phenolic acids in Myrcia bella cambess. Using FIA-ESI-IT-MSn and HPLC-PAD-ESI-IT-MS combined with NMR. Molecules 2013; 18: 8402-16.
2. Wang Y, Yang L, He YQ, Wang CH, Welbeck EW, Bligh SA, et al. Characterization of fifty‐one flavonoids in a Chinese herbal prescription Longdan Xiegan Decoction by high‐performance liquid chromatography coupled to electrospray ionization tandem mass spectrometry and photodiode array detection. Rapid Communications in Mass Spectrometry: An International Journal Devoted to the Rapid Dissemination of Up‐to‐the‐Minute Research in Mass Spectrometry 2008; 22: 1767-78.
3. Fabre N, Rustan I, de Hoffmann E, Quetin-Leclercq J. Determination of flavone, flavonol, and flavanone aglycones by negative ion liquid chromatography electrospray ion trap mass spectrometry. Journal of the American Society for Mass Spectrometry 2001; 12: 707-15.
4. Wu W, Yan C, Li L, Liu Z, Liu S. Studies on the flavones using liquid chromatography–electrospray ionization tandem mass spectrometry. Journal of Chromatography A 2004; 1047: 213-20.
5. Li H, Wan L, Hashi Y, Chen S. Fragmentation study of a 8‐C‐glycosyl isoflavone, puerarin, using electrospray ion trap time‐of‐flight mass spectrometry at high resolution. Rapid Communications in Mass Spectrometry: An International Journal Devoted to the Rapid Dissemination of Up‐to‐the‐Minute Research in Mass Spectrometry 2007; 21: 2497-504.
6. Choi JY, Lee SJ, Lee SJ, Park S, Lee JH, Shim JH, et al. Analysis and tentative structure elucidation of new anthocyanins in fruit peel of Vitis coignetiae Pulliat (meoru) using LC‐MS/MS: Contribution to the overall antioxidant activity. Journal of separation science 2010; 33: 1192-7.
7. Riethmüller E, Tóth G, Alberti Á, Végh K, Burlini I, Könczöl Á, et al. First characterisation of flavonoid-and diarylheptanoid-type antioxidant phenolics in Corylus maxima by HPLC-DAD-ESI-MS. Journal of pharmaceutical and biomedical analysis 2015; 107: 159-67.
8. Ren X, He T, Chang Y, Zhao Y, Chen X, Bai S, et al. The genus Alnus, a comprehensive outline of its chemical constituents and biological activities. Molecules 2017; 22: 1383.
